# Influence of virtual reality on visual parameters: immersive versus non-immersive mode

**DOI:** 10.1186/s12886-020-01471-4

**Published:** 2020-05-24

**Authors:** Hyeon Jeong Yoon, Jonghwa Kim, Sang Woo Park, Hwan Heo

**Affiliations:** Department of Ophthalmology, Chonnam National University Medical School and Hospital, 42 Jebong-ro, Dong-gu, Gwangju, 61469 Republic of Korea

**Keywords:** Accommodation, Asthenopia, Convergence, Virtual reality

## Abstract

**Abstract:**

**Background:**

To investigate the differences in refraction, accommodative factors, visual parameters, and subjective symptoms after using two types of virtual reality (VR) content with different depths of perception.

**Methods:**

Twenty-three volunteers, who played VR games in two modes (immersive and non-immersive) for 30 min, were enrolled. Visual parameters were examined before and after using VR. Accommodative factors were measured using static and dynamic methods. Subjective symptoms were assessed using a questionnaire. Differences according to VR content were compared, and correlations between each visual parameter were analyzed.

**Results:**

There were no changes in refraction or accommodative factors after use of the VR. However, there was a significant increase in the near point of accommodation (NPA), the near point of convergence (NPC), and subjective symptom scores after using the immersive mode. Correlation analysis revealed a positive correlation between baseline values of near exophoria and mean accommodative lag of the dominant eye, and also revealed a negative correlation between NPA and mean accommodative lag in the non-dominant eye.

**Conclusions:**

The use of VR for 30 min increased NPA and NPC, especially after the immersive mode was used. In addition, higher exophoria and smaller NPA is associated with increased accommodative lag after using VR.

## Background

A virtual reality (VR) device is an immersive medium that uses a head-mounted display (HMD). VR creates the sensation of being entirely transported into a virtual three-dimensional (3D) world, which can provide a far more visceral experience compared to other video formats. Recently, VR devices have become available for purchase on the Internet from a variety of manufacturers, and have been widely used for gaming.

However, the proper use of VR has not yet been established. In health and safety warnings provided by manufacturers, VR is not recommended for use by children < 13 years of age. Additionally, continuous use for > 30 min is discouraged, although these warnings are based on weak evidence [1]. Many studies have reported that a significant proportion of VR device users experience a highly aversive sense of discomfort, disorientation, nausea, and motion sickness, and these reports suggest that viewing stereoscopic images on 3D devices may induce visual asthenopia, such as visual discomfort and fatigue [2–6]. It is essential to study the effects of VR devices on the eye, as HMD images are presented to users at a short distance with a powerful convex lens to simulate 3D reality. Compared with older types of VR, many improvements have been made to reduce user discomfort including image resolution and corresponding processes for head movement. However, there are few ophthalmological studies investigating the effects of recent iterations of VR devices [7–11].

Ha et al. [7] investigated the clinical effects of the HMD on visual function, including the oculomotor system; they found no significant clinical changes, except for transient refractive error or binocular alignment. However, the study adopted the method of watching movies using VR rather than immersive content such as games. In that case, the perceived depth was fixed at one distant point; therefore, it is possible that visual parameters change and the accommodation-convergence conflict is not fully induced, compared with immersive content, which has variable perceived depth. In addition, accommodative change was not evaluated. It is necessary to investigate accommodative change, as it can be related to a user’s transient or permanent myopia, in addition to subjective symptoms [12–14].

Turnbull et al. [15] reported that refraction and binocular status (e.g. gaze stability, stereopsis, and amplitude of accommodation) did not change after VR trials. However, the choroid, which is a pigmented vascular tissue located outside of the eye, was thickened. In that study, investigators used virtual indoor and outdoor environmental content. They also examined accommodative amplitudes, but they did not analyze their correlation with other visual parameters.

In the present study, we examined changes in objective visual parameters and subjective symptoms after playing two modes of VR content – immersive and non-immersive – each with a different perception depth. The change in accommodation was evaluated using static and dynamic methods. In addition, correlations between visual parameters after playing VR content were evaluated.

## Methods

Thirty-five healthy volunteers who had better than 16/20 uncorrected visual acuity with above 20/20 best-corrected visual acuity were recruited. The subjects had no ophthalmologic diseases, including strabismus, amblyopia, corneal or retinal disease, or a history of ocular surgery, except for refractive surgery. The number of subjects was calculated using G-power version 3.1 (Heinrich Heine University, Dusseldorf, Germany) and considered a drop-out rate of 20%. Twelve subjects with exophoric deviation > 10 prism diopters (PD) and/or esophoric deviation > 5 PD were excluded. Informed consent was obtained from all 23 volunteers who were eligible and ultimately enrolled in this study. Ethics committee approval was obtained from the Chonnam National University Hospital Institutional Review Board (Gwangju, Korea). The study protocol adhered to the guidelines of the Declaration of Helsinki.

### Display

The Oculus Rift VR device (Oculus VR, LLC., Irvine, California, USA) was used in this study. The device comprised a lightweight (0.44 kg) headset that completely covered the field of view. The headset included separate displays for each eye, each with 960 × 1080 resolution, yielding a 100-degree-horizontal field of view. A fixed-degree convex lens was located in front of each display rendered display content at optical infinity. Inter-pupillary distance was adjusted via a user-enabled key that was located on the right side of the VR device.

Participants used the Oculus Rift device while seated on a freely rotating chair. They were asked to perform 30 min of gameplay (Minecraft, Mojang AB, Sweden) in two different modes (immersive and non-immersive). There was a 1-week interval between playing in immersive mode and non-immersive mode. In the immersive mode, the stereo head-tracking head-mounted display presentation brought the player inside the 360-degree virtual reality environment, which allowed the user to feel as if they were physically present in the game. The viewpoint moves in accordance with the player’s head movements. In the non-immersive mode, the player is placed in a static environment (e.g., a living room) while watching the VR environment on a desktop screen that was approximately 2 m away (a desktop view). The players could look around the room; however, the area of gameplay was fixed on a virtual screen in front of the player (Additional file 1).


**Additional file 1:** Immersive vs. non-immersive mode (AVI). Video clip showing the difference between immersive and non-immersive modes. In the non-immersive mode, the viewing angle is fixed parallel to the ground, while in the immersive mode, the view is free to rotate.


### Measurements of accommodation

Refraction and accommodation were measured using a binocular open-field refractor (Auto Ref/Keratometer WAM-5500, Grand Seiko Co Ltd., Hiroshima, Japan). The spherical equivalent (sphere + 1/2 of cylinder) was used for calculation. For static measurement, the accommodative amplitude was calculated by subtracting the refractions obtained under monocular condition while viewing a 1 cm × 1 cm E-shape target at 33 cm from those obtained from viewing the target at 5 m in the same manner.

Software verified by the manufacturer was installed on a computer to allow dynamic mode function. To initiate measurements, the instrument was aligned with the pupil of each eye, and the joystick button was pushed and then released once; the instrument then commenced recording dynamic measurements at approximately 5 samples/s. The observer ensured that the instrument remained carefully aligned with the subject’s right eye while undergoing dynamic measurements by observing the alignment target imaged within the pupillary center in the LCD monitor for the entire duration of testing. The instrument wrote the data to a spreadsheet file (Excel, Microsoft Corporation, Redmond, WA, USA) that recorded the time of measurement, eye measured, spherical equivalent refraction, and pupil diameter approximately every 0.2 s and converted it to a sine graph form [16, 17]. The velocity of accommodation, mean accommodative lag, and dynamic accommodative response was investigated to measure dynamic accommodation.

**Fig. 1:**
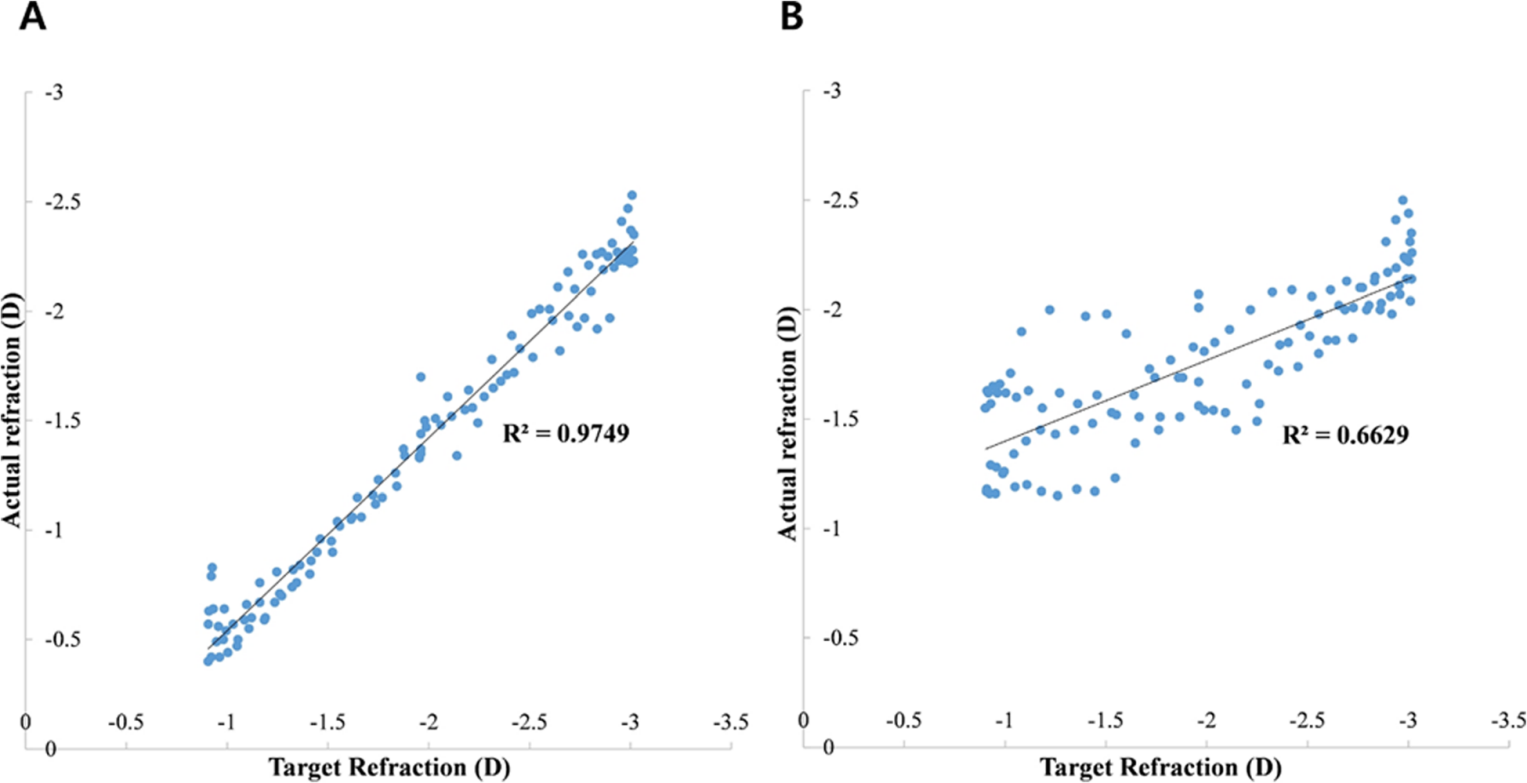
Dynamic accommodative response was calculated according to the dispersion between actual refraction and target refraction (**a** demonstrates a better dynamic accommodative response than **b**)

The velocity of accommodation was obtained by calculating the difference between the maximum and minimum refraction divided by the time taken. Mean accommodative lag was calculated by averaging the value of the participant’s actual refraction that differed from the target refraction. The dynamic accommodative response was calculated according to the dispersion between actual refraction and target refraction. A higher correlation coefficient was associated with better dynamic accommodative response (Fig. 1).

### Other visual parameters

Monocular near point of accommodation (NPA) was obtained using Donder’s push-up method. A 20/30 single letter about 50 cm from the subject on a fixation stick served as the target, and was moved gradually closer to the participant at a rate of about 5.0 cm/s until the participant noticed the target starting to blur. The near point of convergence (NPC) was also obtained using the same method as previously described for the NPA measurement. The first point at which the corneal reflex of the subjects began to extend outward was considered to be the endpoint [2].

Near stereopsis was measured using a near stereopsis vision test (Stereo Fly SO-001 test; Stereo Optical Co., Chicago, IL, USA). The test stereogram was held 40 cm from the subject during the test. Threshold stereopsis level was recorded in seconds of arc.

Ocular dominance was determined by the hole-in-the-card test. The participant was asked to hold a card with a hole at arm’s length and focus on a target 3 m away with both eyes. The examiner alternately occluded eyes to determine which eye was viewing the target through the hole and that eye was determined to be the dominant eye [18].

The presence and magnitude of far (5 m) and near (33 cm) phoria were verified using the cover test and alternating cover test with prism. A standard set of loose plastic prisms was used, which individual prisms increased in power from 1 to 10 PD in 1-PD increments, and from 10 to 20 PD in 2-PD increments. All measurements were repeated three times for each test, and results reported as the mean value [2].

All measurements were performed before and immediately after playing the VR game in the order listed above. If visual parameters were changed, it was measured repeatedly every 15 min until the initial value was obtained again. The criteria for re-examination were > 2-cm changes in NPA and NPC, over 20 s of arc change of stereopsis, and over 0.5 D change of refraction. Ocular phoric deviation was evaluated by the same pediatric ophthalmologist (H.H.). The other visual parameters were examined by a single examiner (H.J.Y.).

### Evaluation of subjective symptoms

Thirteen symptoms were included in the questionnaire. The questionnaire was based on a computer vision syndrome questionnaire previously described by Seguí del M et al. [19]. The symptom sensation questionnaire contained six identical analog scales (0 = none, 6 = too severe to tolerate) through which the subject recorded the magnitude of each of the symptoms compared with baseline. After playing two modes of the VR game, the subjects completed the questionnaire.

### Statistical analysis

Statistical analysis was performed using SPSS version 18.0 (IBM Corporation, Armonk, NY, USA) for Windows (Microsoft Corporation, Redmond, WA, USA). The normal distribution for all variables was assessed using the Kolmogorov-Smirnov test. All variables were not normally distributed. Data are presented as the median (interquartile range). A Wilcoxon signed-rank test was used to compare changes in variables before and after performing VR. Differences in subjective symptoms according to the contents were also compared using the Wilcoxon signed-rank test. Spearman’s rho correlation test between each of the visual parameters was used for correlation analysis. The variables for a single eye, including NPA and accommodative parameters, were solely correlated with the corresponding eye. For all tests, statistical significance was determined to be *p* < 0.05. with differences corrected by the Benjamini-Hochberg procedure using false discovery rates of 0.25.

## Results

Among the 23 participants, 11 were men and 12 were women, with a mean age of 23.9 ± 3.7 years (range, 20–35 years). The mean uncorrected visual acuity (logarithm of the minimum angle of resolution [logMAR]) was 0.03 ± 0.04 logMAR. Fifteen of the participants had previous experience with VR within 1 h, which was outside of this experiment. Eleven of the participants had a history of refractive surgery for myopia. One participant was excluded from the analysis after discontinuing the immersive mode of VR due to severe headache and nausea.

In the immersive mode, the mean refractive error of both eyes did not change significantly (*p* = 0.935 in the dominant eye; *p* = 0.654 in the non-dominant eye). However, the NPA was increased in both eyes (*p* = 0.005 in the dominant eye, *p* = 0.002 in the non-dominant eye). The NPC was also increased in the immersive mode (*p* = 0.001). In the non-immersive mode, the mean refractive error did not change significantly for either eye (*p* = 0.261 in the dominant eye; *p* = 0.881 in the non-dominant eye). Only the NPC (*p* = 0.002) was increased after using VR. Near stereopsis and phoria were not significantly different in either mode (Table 1).

**Table 1:** Comparison of changes in visual parameters after playing a virtual reality game with different depths perception

Variable	Pre	Post	***p-***value
**Immersive mode**			
Refraction (dominant eye), Diopter	−0.38 (0.63)	− 0.25 (0.50)	0.935
Refraction (non-dominant eye), Diopter	−0.19 (0.81)	−0.13 (0.81)	0.654
NPA (dominant eye), cm	8.50 (3.00)	10.0 (3.50)	0.005*
NPA (non-dominant eye), cm	8.50 (3.00)	10.0 (3.50)	0.002*
NPC, cm	7.00 (3.00)	9.00 (3.25)	0.001*
Near stereopsis, sec	40.0 (12.50)	40.0 (12.50)	0.180
Phoria, PD (near)	2.00 (8.00)	2.00 (8.00)	0.086
**Non-immersive mode**			
Refraction (dominant eye), diopter	−0.38 (0.63)	−0.25 (0.63)	0.261
Refraction (non-dominant eye), diopter	−0.25 (0.88)	−0.25 (0.63)	0.881
NPA (dominant eye), cm	9.00 (3.00)	9.50 (4.25)	0.058
NPA (non-dominant eye), cm	9.50 (3.00)	9.00 (4.00)	0.120
NPC, cm	8.00 (3.00)	9.00 (4.00)	0.002*
Near stereopsis, sec	40.0 (10.0)	40.0 (20.0)	0.234
Phoria, PD (near)	5.00 (8.00)	5.00 (8.00)	0.257

Data presented as median (interquartile range). NPA, near point of accommodation; *NPC* near point of convergence; *PD* prism diopter *statistically significant value using the Benjamini-Hochberg procedure

Table 2 summarizes the comparisons between subjective symptoms according to the immersive and non-immersive VR modes. Tearing, blurred vision, double vision, difficulty focusing for near vision, and neurological symptoms, including headache, dizziness and nausea were more severe in the immersive mode than the non-immersive mode of VR (all *p* < 0.05). Table 3 summarizes the changes in accommodation using static and dynamic measurements with the WAM-5500 binocular refractor after using VR. There was no significant change in accommodative amplitude, velocity of accommodation, mean accommodative lag, or dynamic accommodative response in both modes.

**Table 2:** Comparison of subjective symptoms after playing a virtual reality game

Symptom	Immersive mode	Non-immersive mode	***p***-value
Burning	0.0 (0.0)	0.0 (0.0)	0.564
Feeling of a foreign body	0.0 (0.0)	0.0 (0.0)	0.705
Excessive blinking	1.0 (3.0)	1.0 (2.0)	0.554
Tearing	0.0 (1.0)	0.0 (0.0)	0.014*
Dryness	0.0 (3.0)	1.0 (2.0)	0.942
Tingling	0.0 (2.0)	0.0 (1.0)	0.084
Blurred vision	1.0 (2.0)	0.0 (1.0)	0.005*
Double vision	0.0 (0.0)	0.0 (0.0)	0.046*
Difficulty focusing for near vision	0.0 (1.0)	0.0 (0.0)	0.018*
Increased sensitivity to light	0.0 (1.0)	0.0 (0.0)	0.623
Headache	2.0 (3.0)	0.0 (1.0)	0.012*
Dizziness	3.0 (3.0)	1.0 (2.0)	0.012*
Nausea	3.0 (3.0)	0.0 (1.0)	0.004*
**Total**	12.0 (9.0)	5.0 (10.5)	0.002*

Data presented as median (interquartile range). *statistically significant value using the Benjamini-Hochberg procedure

**Table 3:** Changes in accommodation using static and dynamic measurement after playing a virtual reality game

Variable	Immersive mode			Non-immersive mode		
	Pre	Post	*p*-value	Pre	Post	*p*-value
**Static measurement – Accommodative amplitude, diopter**						
Dominant eye	2.063 (1.188)	1.938 (0.688)	0.722	2.000 (0.500)	1.875 (0.875)	0.571
Non-dominant eye	2.063 (1.375)	2.125 (1.313)	0.442	2.000 (0.875)	2.000 (1.000)	0.572
**Dynamic measurement**						
**Velocity of accommodation, diopter/s**						
Dominant eye	0.334 (0.126)	0.395 (0.144)	0.095	0.382 (0.105)	0.361 (0.154)	0.910
Non-dominant eye	0.416 (0.104)	0.383 (0.119)	0.664	0.364 (0.133)	0.358 (0.133)	0.362
**Mean accommodative lag, diopter**						
Dominant eye	0.660 (0.628)	0.602 (0.483)	0.108	0.575 (0.520)	0.591 (0.528)	0.322
Non-dominant eye	0.707 (0.430)	0.836 (0.590)	0.274	0.734 (0.357)	0.680 (0.489)	0.548
**Dynamic accommodative response**						
Dominant eye	0.912 (0.078)	0.901 (0.101)	0.778	0.884 (0.105)	0.904 (0.126)	0.664
Non-dominant eye	0.881 (0.112)	0.900 (0.150)	0.821	0.914 (0.057)	0.886 (0.101)	0.099

Data presented as median (interquartile range).

Table 4 summarizes the correlations between the baseline data of visual parameters (exophoria at far/near, NPA in the dominant/non-dominant eye and NPC), changes in ocular and accommodative parameters, and the sum of subjective symptom scores after using VR. There was a positive correlation between baseline values of near exophoria and mean accommodative lag of the dominant eye (r = 0.372, *p* = 0.014). The NPA in the non-dominant eye exhibited a negative correlation with changes in mean accommodative lag of the dominant eye (r = − 0.328, *p* = 0.032). Two correlation measurements were excluded due to a false positive rate above 0.3: exophoria versus changes of the NPA and the NPC versus changes of mean accommodative lag.

**Table 4:** Correlations between the value of baseline and changes of ocular parameters

Changes in value (post - pre)	Exophoria (far)		Exophoria (near)		NPA(dominant eye)		NPA (non-dominant eye)		NPC	
	r	*P*	r	*P*	r	*P*	r	*P*	r	*P*
**Ocular parameters**										
Exophoria (far)	−0.100	0.514	0.127	0.410	−0.032	0.841	− 0.064	0.685	−0.087	c
Exophoria (near)	− 0.166	0.276	− 0.149	0.335	− 0.054	0.731	−0.066	0.676	−0.152	0.324
NPA (dominant eye)	0.296	**0.048**	−0.007	0.963	−0.255	0.099			0.007	0.966
NPA (non-dominant eye)	0.202	0.184	−0.028	0.859			−0.204	0.189	0.105	0.498
NPC	0.046	0.764	0.046	0.764	0.051	0.747	0.051	0.747	−0.188	0.222
**Accommodative parameter (dominant eye)**										
Accommodative amplitude	−0.110	0.507	0.030	0.860	−0.220	0.191			−0.003	0.987
Velocity of accommodation	0.059	0.703	−0.034	0.834	0.123	0.438			0.001	0.994
Mean accommodative lag	0.165	0.285	0.372	0.014*	−0.175	0.266			−0.159	0.309
Dynamic accommodative response	0.032	0.845	0.139	0.393	0.085	0.607			0.004	0.979
**Accommodative parameter (non-dominant eye)**										
Accommodative amplitude	−0.141	0.373	−0.034	0.834			−0.128	0.430	−0.008	0.959
Velocity of accommodation	−0.017	0.913	0.151	0.327			−0.005	0.976	0.207	0.187
Mean accommodative lag	0.111	0.468	0.076	0.624			−0.328	0.032*	−0.333	**0.027**
Dynamic accommodative response	−0.110	0.507	0.019	0.903			0.003	0.983	−0.069	0.659
**Sum of symptom scores**	−0.110	0.471	0.173	0.261	0.198	0.202	0.222	0.152	0.084	0.587

*NPA* near point of accommodation, *NPC* near point of convergence *statistically significant value using the Benjamini-Hochberg procedure

No visual parameters were found that directly correlated with symptoms. There was no correlation between total symptom score and baseline value of visual parameters (all *p* > 0.05; Table 4). This result was the same as the analysis using logistic regression (all p > 0.05). Additionally, we examined the correlations between visual parameters and each of the subjective symptoms, and there were no statistically significant correlations (all p > 0.05).

## Discussion

VR is a technology that renders a proximal display to be perceived as a real-world experience using powerful convex lenses. In a VR environment, accommodation is fixed to a single depth of field at a distant point. However, convergence is constantly induced. The resulting accommodation-convergence conflict and sustained eyeball movement are known to cause fatigue and 3D asthenopia [8, 20–22].

In this study, the NPA and NPC increased in the immersive mode. In the immersive mode, more image disparity can occur than in the non-immersive mode. Image disparity activates an accommodative response and convergence-accommodation to a change in accommodation [23–25]. However, the actual accommodative target was fixed; thus, there was a possibility of fatigue due to the excessive activation of accommodative adaptation [24]. As shown in Table 3, other accommodative factors did not change. It appears that ocular fatigue induced an increase in the NPA and NPC, which is more of a subjective factor than actual accommodation.

The data presented in Table 2 demonstrate that in the immersive mode, neurological symptoms, such as headache, dizziness and nausea, were more severe than discomfort in the eyes such as dryness. In addition, blurred vision, double vision, and defocusing symptoms were worse in the immersive mode. These results suggest that ocular fatigue due to excessive accommodation-convergence response is more severe in the immersive mode.

As shown in Table 4, correlation analysis revealed that higher exophoria is associated with increased accommodative lag. Vergence adaptation modulates fast response and reduces error and fatigue by maintaining vergence stimulus [23–25]. Exophoric subjects may require more effort in accommodation and convergence due to reduced adaptation, which can cause more fatigue than in normal individuals [4, 26–29].

In addition, subjects who had a smaller NPA were more likely to exhibit an increase in mean accommodative lag after using VR. The baseline NPC was removed due to a high false discovery rate; however, the baseline NPC showed a relatively high correlation with changes in mean accommodation lag. Although not included in the table, the baseline NPA and NPC demonstrated a high positive correlation with baseline values of mean accommodative lag (dominant eye, *p* = 0.010, *p* = 0.008; non-dominant eye, *p* = 0.026, *p* = 0.017, respectively).

Sreenivasan et al. [30] reported that retinal image quality is better when accommodative lag is greater, because, paradoxically, the depth of field is more structurally or functionally wider in individuals with higher accommodative lag. In individuals with a large baseline NPA and NPC, retinal image quality was paradoxically better, as with high accommodative lag. This may reduce the ocular fatigue that is induced by the rapid accommodation response.

However, there is controversy regarding whether accommodative lag enhances accommodative stress by promoting a blurred retinal image [31]. Further research is needed to support this hypothesis. Shiomi et al. [32] reported that actual accommodation could change depending on the perception depth of the 3D content, even when the 3D display is fixed. Unlike previous reports, the use of VR appears to affect accommodation, and further studies are needed to resolve these issues.

Turnbull et al. [15] suggested that increased choroidal thickness caused by myopic retinal defocus could be associated with reduced myopia progression. In our study, myopic shift and hyperopic shift > 0.5 D was evident in each of the 3 cases in the immersive mode. In the non-immersive mode, myopic shift and hyperopic shift were observed in 8 cases and 4 cases, respectively. However, these changes fully recovered within 1 h. Our study showed that myopic and hyperopic shifts presented atypically and did not correlate with other ocular factors or VR mode. In addition, a study by Ha et al. [7] reported that transient myopia could occur after using VR. The hypothesis of increased or reduced myopic progression using VR appears to require a more cautious approach.

One limitation of this study was the inclusion of a specific population (i.e., 23 subjects 20 to 35 years of age, with an uncorrected visual acuity of 0.8 or higher). Thus, it is difficult to judge the effect on users of different conditions. Notably, the actual refraction data were not analyzed, including those of subjects with a history of refractive surgery. It is possible that the use of VR for only 30 min was insufficient to produce changes in visual parameters. Furthermore, there were no control groups that did not use VR. Additional larger-scale studies are needed to resolve these limitations. However, we propose that it may be meaningful that the results of this study demonstrate the difference in visual parameters depending on the contents, despite using the same VR device.

## Conclusion

In conclusion, the use of VR for approximately 30 min did not affect refraction, regardless of the VR mode; however, it increased the NPA and NPC, especially after using the immersive mode. In addition, correlation analysis revealed that higher exophoria and a smaller NPA is associated with increased accommodative lag after using VR. This study was the first to evaluate VR influence, which was assessed using immersive, real 3D content, instead of non-immersive VR content. Additionary, accommodation was analyzed using dynamic techniques. Our results may form the basis for recommendations for users who may need to be more careful regarding VR use.

## Data Availability

Data supporting our findings are contained in the manuscript. However, the raw data set on which the conclusion was made is available on request from Professor Hwan Heo (contact email:opheye@hanmail.net).

## Abbreviations

VR: Virtual reality
NPA: near point of accommodation
NPC: near point of convergence
HMD: head-mounted display
PD: prism diopters
